# Single-session intervention on growth mindset on negative emotions for university student mental health (U-SIGMA): a protocol of two-armed randomized controlled trial

**DOI:** 10.1186/s13063-023-07748-5

**Published:** 2023-11-08

**Authors:** Shimin Zhu, Yuxi Hu, Di Qi, Nan Qin, Xinli Chi, Jiawen Luo, Jie Wu, Hua Huang, Qiaobing Wu, Lu Yu, Shiguang Ni, Kyra Hamilton, Samson Tse

**Affiliations:** 1 Department of Applied Social Sciences, The Hong Kong Polytechnic University, Hong Kong SAR, China; 2 Mental Health Research Centre, The Hong Kong Polytechnic University, Hong Kong SAR, China; 3 School of Public Administration, Guangdong University of Finance & Economics, Guangzhou, China; 4 Institute of Mental Health, School of Psychology, Shenzhen University, Shenzhen, China; 5 Mental Health Education Center, Guangdong University of Technology, Guangzhou, China; 6 Faculty of Education, Zhaoqing University, Zhaoqing, China; 7 Graduate School at Shenzhen, Tsinghua University, Shenzhen, China; 8 School of Applied Psychology, Menzies Health Institute Queensland, Griffith University, Mt Gravatt, QLD Australia; 9 Department of Social Work and Social Administration, Faculty of Social Sciences, The University of Hong Kong, Hong Kong SAR, China

**Keywords:** Belief in change, University students, Common mental health symptoms, Help-seeking

## Abstract

**Background:**

The university years are a developmentally crucial phase and a peak period for the onset of mental disorders. The beliefs about the changeability of negative emotion may play an important role in help-seeking. The brief digital growth mindset intervention is potentially scalable and acceptable to enhance adaptive coping and help-seeking for mental health needs in university students. We adapted the Single-session Intervention on Growth Mindset for adolescents (SIGMA) to be applied in university students (U-SIGMA). This protocol introduces a two-armed waitlist randomized controlled trial study to examine the effectiveness and acceptability of U-SIGMA in promoting help-seeking among university students in the Greater Bay Area.

**Methods:**

University students (*N* = 250, ages 18–25) from universities in the Greater Bay Area will be randomized to either the brief digital growth mindset intervention group or the waitlist control group. Participants will report on the mindsets of negative emotions, perceived control over anxiety, attitude toward help-seeking, physical activity, hopelessness, psychological well-being, depression, anxiety, and perceived stress at baseline and the 2-week and 8-week follow-ups through web-based surveys. A 30-min digital intervention will be implemented in the intervention group, with a pre- and post-intervention survey collecting intervention feedback, while the control group will receive the link for intervention after 8 weeks.

**Discussion:**

This protocol introduces the implementation plan of U-SIMGA in multi-cities of the Greater Bay Area. The findings are expected to help provide pioneer evidence for the effectiveness and acceptability of the brief digital intervention for university students in the Chinese context and beyond and contribute to the development of accessible and effective prevention and early intervention for university students’ mental health.

**Trial registration:**

HKU Clinical Trials Registry: HKUCTR-3012; Registered 14 April 2023. http://www.hkuctr.com/Study/Show/7a3ffbc0e03f4d1eac0525450fc5187e.

**Supplementary Information:**

The online version contains supplementary material available at 10.1186/s13063-023-07748-5.

## Background

University students are vulnerable to developing mental health problems. Anxiety and depression are common among university students, with a 12-month prevalence for generalized anxiety disorder and major depressive episodes being 16.7% and 18.5%, respectively [1]. Suffering from mental health symptoms can profoundly affect university students’ work capacity, academic performance, physical health, and graduation rates, impairing their daily functioning [2, 3]. Therefore, developing effective prevention and early intervention for mental disorders in university students is paramountly needed.

Despite the availability of counseling centers in universities, the majority of anxious and depressed students do not actively seek campus-based interventions, partially due to the concerns of stigma and discrimination [4]. Moreover, even for youths who use health care services, the overall number of visits is just 3.9 on average implying a lack of sustained involvement in therapy [5]. Given the large number of students with mental health care needs and the low engagement in traditional services, there is clearly a need to develop briefer, more scalable, non-stigmatizing, and youth-friendly interventions to promote mental health and help-seeking of university students.

Self-guided brief digital psychoeducation interventions are novel initiatives that are potentially scalable and acceptable to help this important segment of population during a critical life course stage [6]. Digital and web-based interventions are easily adopted by young people, provide more anonymity, and enable remote use when it is not possible for students to access conventional face-to-face psychotherapy [7]. Although extant studies on digital intervention have yielded promising results [8, 9], only a limited number of interventions were designed based on the university students’ needs [10]. Brief digital intervention can prompt changes if the design of the intervention is relevant to meaning-making and social context [11].

Growth mindsets, the beliefs in change of personal attributes, are closely related to psychopathology [12], and the mindset intervention has recently been applied to clinical practice [13]. Fixed mindsets are linked to more suppressive emotion regulation and medication therapy choice while growth mindsets are associated with cognitive reappraisal and effort-taking therapy choices, such as counseling [14, 15]. In addition, growth mindsets alleviate the associations between stressful life events and mental distress [16]. The growth mindset interventions have shown promising effects [13, 17], particularly in enhancing perceived emotional control and decreasing anxiety symptoms [18]. However, single-session growth mindset intervention is far from sophisticated and needs further refinement and delicate design for the targeted population. Furthermore, as the effects of single-session intervention may wane over time, booster messages should be adopted to strengthen the efficacy [19]. The current study is to tailor-design a brief digital growth mindset intervention for Hong Kong and mainland Chinese university students based on the extant research findings, including those from overseas and local empirical research.

Previous studies have found that the mindset regarding negative emotions is a key predictor for self-harm, suicidality, social withdrawal [20], gaming disorder [21], depression, and anxiety [22, 23]. Mindsets were also found to mediate the association between depression and suicidality, that is, individuals suffering from depression are less likely to develop suicidal ideation if they have higher growth mindsets [24]. Besides, growth mindsets predict adaptive emotion regulation (e.g., sports) and higher subjective well-being of individuals under adversities [25]. These findings provide strong empirical support that fostering belief-in-change of negative emotions is likely to promote mental health among university students. Based on the findings from these studies, a *S*ingle-session *I*ntervention of *G*rowth *M*indset for *A*dolescents (SIGMA) was designed to examine its effectiveness among secondary school students [26].

Building on our previous research, we have adapted the SIGMA growth mindset intervention and developed a brief digital intervention with boosters for university students, i.e., the U-SIMGA. We included examples relevant to university life and adapted the engaging exercise in the U-SIMGA to match the cognitive ability with university students. This proposed study will conduct a randomized controlled examination on the acceptability and effectiveness of the intervention compared to a waitlist control. University students in Hong Kong and other cities in the Greater Bay Area of Mainland China (mainly from Guangzhou, Shenzhen, and Zhaoqing) are invited to join the study as many universities located in this area and the widespread use of digital devices among university students. The proposed study aims to examine the effectiveness and acceptability of the brief digital growth mindset intervention in promoting university students’ mental health, specifically in [1] enhancing the perceived emotional control, help-seeking intention, psychological well-being, and physical activity and [2] reducing symptoms of anxiety, depression, and stress, and hopelessness. Through the study, we will collect empirical evidence and feedback on the acceptability and effectiveness of single-session interventions for Chinese university students. Based on the findings, further improvement can be made to promote effective and scalable brief digital intervention for university students’ mental health.

### Objectives

The primary objective is to evaluate the effectiveness of a brief digital intervention of growth mindsets on negative emotions in the primary outcome (enhancing perceived control over anxiety) and secondary outcomes (i.e., enhancing attitude toward help-seeking, psychological well-being, and physical activity, and reducing depression, anxiety, stress, and hopelessness) in university students. The secondary objective is to evaluate the acceptability of the brief digital intervention by intervention feedback measures among university students in the Greater Bay Area.

### Hypotheses

We hypothesize that the brief digital growth mindset intervention is effective in the primary outcome of (i) enhancing perceived control over anxiety and secondary outcomes of (ii) increasing attitude toward help-seeking, (iii) reducing hopelessness, (iv) enhancing psychological well-being, (v) increasing physical activities, and (vi) reducing depressive, anxiety, and stress symptoms. Moreover, the effectiveness is hypothesized to be greater among participants with higher anxiety and depression symptom, or higher motivation at changing current emotional status and emotion coping than among those with low or no symptoms or motivation.

## Methods

### Study design

**Fig. 1:**
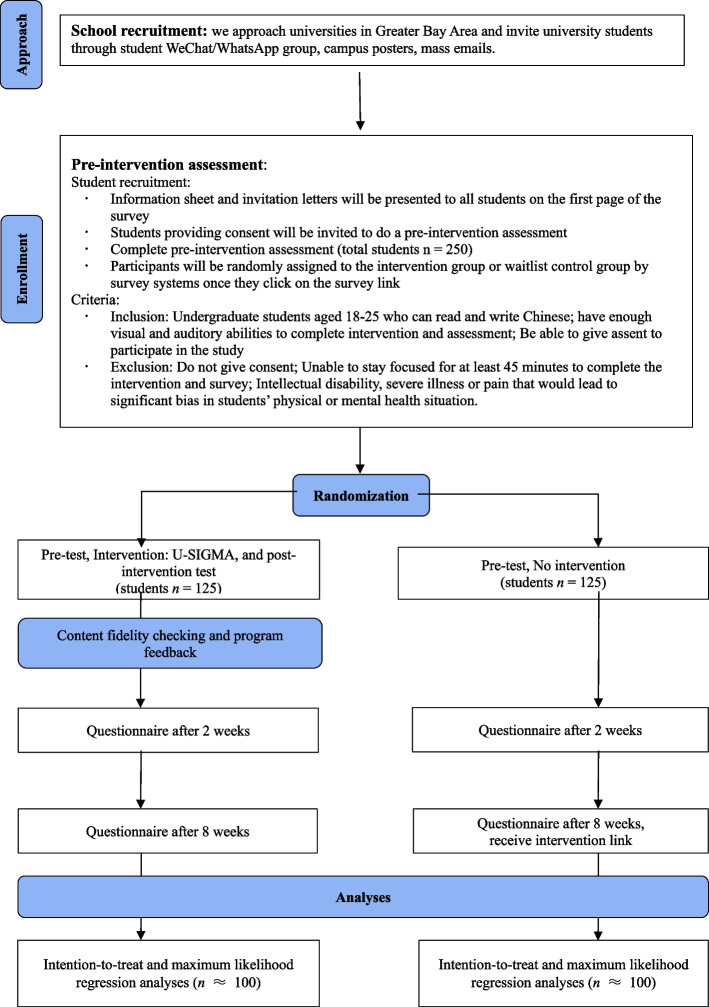
CONSORT diagram reflecting the flow of participants through the current study

For this randomized controlled trial study, invitations for participation will be sent to the teachers of six universities in the Greater Bay Area, and then they will be asked to forward the invitation to WhatsApp or WeChat student groups. We also recruit through mass email and promotion posters. Students who click on the research link will be invited to read the research information sheet and sign the consent form, and the survey will immediately end for those students who do not agree to participate in the study without signing the consent. *Qualtrics* in Cantonese and *WenJuanXing* survey tool in Mandarin will be used for Hong Kong students and Mainland students respectively. Participants will be randomized into the intervention group or the waitlist control group by adding a “Randomizer” block into the survey flow of two online survey platforms. The expected allocation ratio and concealment can be ensured since the “Randomizer” block will only appear after the baseline assessments have been completed by the participants and conduct the allocation based on a predetermined ratio. Participants assigned to the intervention group will start the intervention instantly, and those in the control group will receive the intervention link after 8 weeks. Three repeated assessments of the measures will be conducted for two groups simultaneously at (i) baseline, (ii) 2-week post-intervention, and (iii) 8-week post-intervention via survey systems *Qualtrics* (for Hong Kong students) and *WenJuanXing* (for mainland students). The design of 2-week and 8-week post-tests is based on the extant studies on single-session or brief intervention [12, 27]. Invitations for follow-up surveys will be sent to the participants who complete the survey at baseline. Each participant will receive incentives (e-voucher or online red packet) with approximate values of US $5 and US $8 when completing the 2-week and 8-week follow-up questionnaires respectively. Thus, the total compensation for participation is US $13. Incentives will be sent to the participants automatically through the online system excluding invalid responses after the screening. The study flow is seen in Fig. 1.

### Sample size

The sample size was calculated based on a small-to-medium effect size of *Cohen’s d* = *0.40* from prior research [28], power of 0.80, and alpha of 0.05 (two-tailed). It shows a total of 200 participants (100 per arm) are needed. Referring to the attrition rate of around 20% in our previous school-based studies, it is safe to include 250 participants for this study (125 per arm).

### Participant eligibility

Eligible participants are university students (years 1 to 4) from six universities recruited through cluster randomized sampling. The inclusion criteria are as follows: [1] age 18–25; [2] Chinese students with the ability of reading and writing Chinese; [3] enough visual and auditory abilities; and [4] giving consent for participation. Exclusion criteria include the following: [1] no consent; [2] unable to stay focused for at least 45 min for completion of the intervention and survey; [3] intellectual disabled, suffering from severe illness or pain which could result in biased physical or mental health situation of students.

### Brief digital growth mindset intervention and waitlist control group

**Fig. 2:**
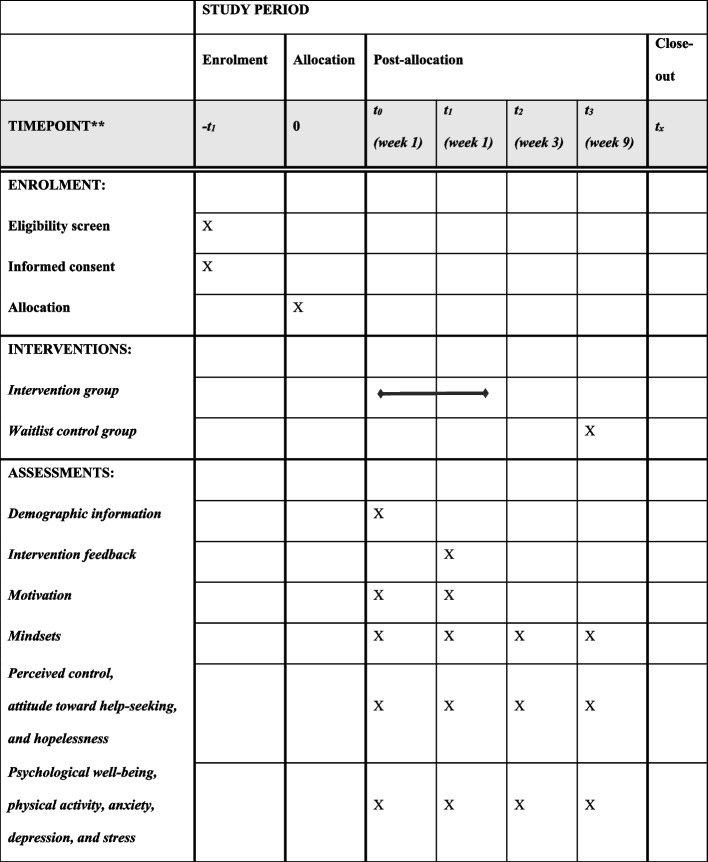
Schedule of enrolment, intervention, and assessments

The randomized controlled trial is designed to demonstrate the superiority of the intervention group to the waitlist group with a 1:1 allocation. The intervention is a web-based intervention, which is adapted from the single-session growth mindset intervention (SIGMA) for secondary school students developed by Zhu et al. [26]. The current intervention adapted the SIGMA intervention content for university students (U-SIGMA) and incorporated boosters to strengthen the effectiveness of the intervention. The U-SIGMA contains five components: (a) introduction of emotional brain to communicate scientific knowledge on emotion and growth mindset regarding emotion, (b) stories and testimonials from university students describing how growth mindsets or fixed mindsets affect their coping with mental health symptoms, (c) emotion coping strategies, (d) common misconceptions and doubts of growth mindset, and (e) self-persuasion writing exercises for participants to share notes on the negative emotion growth mindset with others. Boosters with key messages of intervention are made through e-posters, which will be sent to all participants in the intervention group as reminders weekly from the second week after the intervention through email, WhatsApp, or WeChat, with a total of four times. Besides, boosters will be designed based on the “saying is believing” mechanism, empowering and encouraging the participants to share what they learned with others for knowledge reflection and application. For example, several blanks will be left for the participants to critically think about and write down their own emotional coping strategies or others’ examples of growth mindset based on daily observation. Participants in the waitlist control group will not receive the intervention program until 8 weeks later, though they will be asked to complete three repeated measures at the same time as the intervention group. Figure 2 presents the Standard Protocol Items: Recommendations for Interventional Trial (SPIRIT checklist) about the schedule of enrolment, interventions, and assessments.

### Implementation strategies

Three strategies are adopted to ensure the comprehensibility, relevance, acceptability, and effectiveness of the self-developed digital intervention. First, we formed an advisory group with two undergraduate students and four graduate students, collecting challenges in universities, testimonials of mindsets, personal understandings about growth mindset, and emotion regulation from the perspective of the target population. It is crucial to continuously reflect on the feedback and suggestions in the trial to ensure the contents of the intervention are context-adaptive, self-relevant, and easily understood for university students. We also interviewed them about the duration, type, and flow of the intervention for further improvement in the setting, making sure the intervention is acceptable for the target population. Second, the study will provide opportunities for participants to understand their personal emotional status by immediately providing a system-generated emotion evaluation report based on their responses, which may enhance the motivation for participation and make them feel more self-relevant. Third, the “saying is believing” mechanism is applied throughout the study. Besides the self-persuasion writing exercises that allow the participants to act as “helpers” to others, they are also encouraged to advocate what they have learned and share the intervention link with other university students in need. Participants will receive four digital poster cards designed with key intervention messages and they will be invited to share the cards to other people.

### Measurements

Assessments will consist of demographic information and three major parts: (1) fidelity checking measures, including mindsets of negative emotions and intervention feedback, which involves Likert-scale rating of the acceptability of the intervention and open-ended questions for feedback; (2) primary outcome measures, including perceived control over anxiety, attitude toward help-seeking, and hopelessness; (3) secondary outcome measures, including the psychological wellbeing, physical activity, depression, anxiety, and stress symptoms. Two attention-checking items are embedded in all assessment points’ surveys to screen careless responding.

### Content fidelity checking

*Mindsets of negative emotions* will be assessed by the well-validated Mindset of Depression, Anxiety, and Stress Scale (MDASS) in Chinese [22]. It involves three subscales (i.e., mindsets regarding depression, anxiety, and stress), and each subscale includes 4 items. There are items such as “*When you have a certain level of depression/anxiety/stress, you really cannot do much to change it*.” Participants will rate the 12 items on a 6-point Likert scale (1 = *strongly disagree* to 6 = *strongly agree*). Higher scores represent that participants less believe in the malleability of negative emotions. Good reliabilities of three subscales were reported with Cronbach’s alphas equaling 0.91, 0.89, and 0.90, respectively [22].

*Intervention feedback* will be measured by the self-developed intervention feedback scale referring to the theoretical framework of acceptability (TFA). Seven aspects are involved for consideration, i.e., affective attitude, intervention coherence, burden, opportunity costs, perceived effectiveness, self-efficacy, and ethicality [29]. Six items are developed for each component except ethicality. Besides, the current scale integrates four items and an open-ended written question from the Program Feedback Scale [30] to measure the intervention’s acceptability. Participants will rate these 10 items on a 5-point scale. One example item is “*How acceptable was the intervention to you?*”.

*Motivation* will be measured by self-developed items. Two items are utilized to measure motivation at changing current emotional status before the intervention, including “*How much do you want to change your current emotional state*” and “*How much do you want to improve your emotion regulation ability*”. It is a 6-point Likert scale from 1 (*not at all*) to 6 (*to a very large extent*). Three items are right after the intervention program, measuring the motivation to apply contents learned from the intervention, including “*After the intervention, I have more confidence in emotion regulation abilities*,” “*After the intervention, I want to apply the emotion regulation strategies*,” and “*After the intervention, I want to improve my emotion regulation abilities*.” Participants will need to rate on a 6-point Likert scale from 1 = *strongly disagree* to 6 = *strongly agree*. Higher scores represent that participants have higher motivation to change current emotional status before the intervention and apply what they have absorbed after the intervention.

### Primary outcome

*Perceived control over anxiety* will be assessed by the Emotion Control subscale of the 15-item Anxiety Control Questionnaire (ACQ-EC) [31]. The subscale has five items (e.g., I can usually put worrisome thoughts out of my mind easily), including one reversed item (i.e., *When I am anxious, I find it hard to focus on anything other than my anxiety*). Participants will rate the items on the scale from 0 = *strongly disagree* to 5 = *strongly agree*. Higher scores on anxiety control were found to be associated with lower levels of anxiety [32]. The Cronbach’s alpha was 0.73 [31].

### Secondary outcomes

*Attitude toward help-seeking* will be measured by five items, including two items from the Attitude toward Seeking Professional Psychological Help Scale (ATSPPH) [33] and three self-developed items. The ATSPPH has good reliability with Cronbach’s alpha equaling 0.72 [33], with a sample item from ATSPPH of “*If I believed I was having a mental breakdown, my first inclination would be to get professional attention*.” Three self-developed items include “*When I encounter difficulties, I will not ask help from teachers*,” “*When I encounter difficulties, I will not ask help from social workers/counselors*,” and “*Professional counseling and treatments can help people improve mental health*.” Participants will need to rate the degree of help-seeking on a 6-point Likert scale, ranging from 1 = very untrue of me to 6 = very true of me.

*Psychological well-being* will be measured by the short version of the 14-item Warwick-Edinburgh Mental Well-being Scale (WEMWBS-14) [34, 35]. Participants will need to rate on a 5-point Likert scale to report general well-being states, ranging from 1 (*none of the time*) to 5 (*all of the time*) on items such as “*I have been feeling optimistic about the future*.” A higher average score of all items represents a higher level of psychological well-being among the participants. Good reliability was reported with Cronbach’s alpha of 0.89 [35].

*Physical activity* will be measured by the Chinese version Global Physical Activity Questionnaire (GPAQ) [36], which involves 16 questions of three domains (i.e., activity at work, for transportation, and for recreation). Three questions in the recreational activity domain are used in this study such as “*Do you do any vigorous-intensity sports, fitness, or recreational (leisure) activities that cause large increases in breathing or heart rate like running or football, for at least 10 min continuously?*”. Participants will be asked to report the frequency and duration of physical activities in a typical week.

*Anxiety symptoms* will be assessed by the Chinese version of the Generalized Anxiety Disorder-7 (GAD-7) scale [37, 38] to measure in the past 2 weeks the frequency of being bothered by anxiety symptoms. Participants will be asked to report the frequency from 0 (*not at all*) to 3 (*nearly every day*) on items such as “*Not being able to stop or control worrying*.” The overall scores of all the 7 items indicate the severity of anxiety symptoms: ranging from 0 to 5 means no anxiety symptom, ranging from 5 to 9 means mild anxiety, ranging from 10 to 14 means moderate anxiety, and ranging from 15 to 21 means severe anxiety. Good reliability was reported with Cronbach’s alpha equaling 0.93 [23].

*Depressive symptoms* will be measured by the Chinese version of the Patient Health Questionnaire-9 (PHQ-9) [39, 40] to assess the frequency in the past two weeks of participants being bothered by depressive symptoms. For example, there is an item “*Feeling down, depressed or hopeless*.” Participants will need to report the frequency from 0 (*not at all*) to 3 (*nearly every day*) on items such as “*Feeling down, depressed or hopeless*.” The overall scores of all the 9 items indicate the depression level: ranging from 0 to 5 means no depressive symptom; ranging from 5 to 9 means mild depression; ranging from 10 to 14 means moderate depression, ranging from 15 to 19 means moderately severe depression, and ranging from 20 to 27 means severe depression. The Cronbach’s alpha was 0.85 [23].

*Stress symptoms* will be measured by the Chinese version of the 4-item Perceived Stress Scale (PSS-4) [41, 42] including items such as “*In the last month, how often have you felt that you were unable to control the important things in your life?*”. Participants will need to report the perceived stress level in the previous month, ranging from 0 (*never*) to 4 (*very often*), including two reversed items ranging from 0 (*very often*) to 4 (*never*). A higher summed score means a higher level of self-perceived stress. The Cronbach’s alpha was 0.83 [43].

*Hopelessness* was measured by the helplessness subscale of the Chinese version Demoralization Scale [44, 45]. Participants will be asked to rate the 4 items such as “*I feel hopeless*” on a 5-point Likert scale from 0 = *strongly disagree* to 4 = *strongly agree*. A higher mean score indicates a higher level of hopelessness. A Cronbach’s alpha of 0.72 was reported in the previous study [44].

*Socio-demographic information* includes an array of participant characteristics and is measured at baseline to examine the variability between groups: gender, age, university name, major, grade, ethnicity, education level of parents, whether having siblings, and perceived socio-economic status.

### Data analyses

An intention-to-treat analysis will be conducted, and multiple imputation will be applied to handle the missing data. Descriptive statistics will be used to demonstrate the socio-demographic and all baseline outcome variables by study arms. Outcome variables will be compared between the arms using *t*-tests, one-way analysis of variance. Generalized estimating equations (GEE) will be used for follow-up data over time. Subgroup analysis will be conducted to test the differences among participants of different gender, motivation, and feedback about the intervention and among participants with different levels of anxiety and depression. Two-level analyses are to be conducted since cluster randomization is used [46]. The group effect, the time effect, and the time-by-group interaction effect on the primary and secondary outcomes will be tested with multilevel regression. A *p*-value of < 0.05 signifies statistical significance, and all statistical analyses will be conducted with SPSS Version 26.

### Data management

All data will be collected through online surveys and automatically saved by the system, resulting in an electronic dataset generated by online survey platforms. A system-generated respondent ID will be assigned to each response of participants and is to be stored in an accessible, safe, confidential manner (e.g., store the dataset in a file with a password which will only be available for researchers to access). All datasets and backup files will be maintained for 3 years after the study is completed.

### Research ethics and dissemination

The Institutional Review Board of the Hong Kong Polytechnic University (HSEARS20230321008) has approved this study. The intervention protocol was pre-registered in the HKU Clinical Trials Registry (ref: HKUCTR-3012), and strictly followed the CONSORT guidelines [47, 48]. Participants will be asked to read and sign the digital informed consent before participation. The consent will be automatically obtained and saved in the online system, which allows researchers to download and examine. Participants will voluntarily join the study and have the right to drop out at any time without any punishment or need to seek permission. All the responses and personal information are highly confidential, which will be accessed by a password that only researchers know and be destroyed 3 years after the study ends. This study will not cause any harm, and participants are indicated to seek help from school counselors or teachers if having any discomfort. They can also get further support from the additional resources for referral services provided at the end of the survey. We will not report individual data or identifiable information of the participants.

The trial results will be communicated to participants, healthcare professionals, and the public via publication. The full protocol can be accessed through the Open Science Framework (OSF). We will not grant public access to the participant-level dataset due to confidentiality of participants’ personal information, and statistical code can be obtained upon request.

### Protocol amendments

A formal protocol amendment will be required if the modifications to the protocol may significantly affect the conduct of the study and participants’ benefits or rights. Such amendment aspects include changes in study objectives, study design, target population, sample sizes, procedure, and implementation strategies on intervention, etc., which will seek approval from the Institutional Review Board of the Hong Kong Polytechnic University (IRB) before the implementation. Minor modifications that would not affect the conduct of the study, such as corrections or clarifications to the protocol, will also be documented and notified to IRB for further approval. Trail registry will be notified for any protocol amendments as well.

## Results

Recruitment starts in April 2023. We expect to complete data collection before November 2023 and make the results available in 2024. The publications are expected to be available in 2024.

## Discussion

This study is to evaluate a randomized trial of a brief digital growth mindset intervention on enhancing Chinese university students’ help-seeking and sense of control over mental health in the Greater Bay Area. Previous research provided strong empirical evidence about the association between growth mindsets regarding negative emotions and mental health [23–25], yet no intervention study provide supports on its effectiveness on promoting mental health among university students. Besides, the single-session brief digital intervention was demonstrated to be effective, which is comparable to interventions that take several months, and the effects exist even after a 9-month follow-up [28]. Therefore, we anticipate that the brief digital growth mindset intervention, which instills the belief-in-change regarding negative emotions, will significantly enhance participants’ perceived control over anxiety and help-seeking tendency, increase psychological well-being, proactive coping, such as physical activity, and reduce hopelessness, depression, anxiety, and stress symptoms. Moreover, the subgroup analysis will help identify the effectiveness and acceptability in specific groups of participants.

## Significance

Scalable intervention for mental health promotion among university students is in pressing need. Brief digital intervention is a novel and promising initiative to address this need, particularly for university students who have full access to digital devices. This study, examining the effectiveness and acceptability of single-session intervention on growth mindset for university students (U-SIGMA), will serve as a building block for the further development of tailored-made brief cost-efficient intervention for university students, contribute to the theoretical and practical progress of digital psychiatry, and bring benefits to the Hong Kong and mainland Chinese university students and beyond.

## Limitations

There are limitations for further improvements. First, this study targets community samples and includes all participants who pass the attention-checking for data analysis. The efficacy of reducing anxiety and depressive symptoms for intervention may not be significant in participants without anxiety or depressive symptoms or with low levels of anxiety and depression in self-report assessments. Nevertheless, sub-group analyses based on the severity of anxiety and depressive symptoms will be conducted. We can also explore the relationship between motivation of participation and the severity of symptoms in sub-groups. Second, due to the unpredictable number of participants from different universities and different cities, the multi-site comparison may not be feasible due to the uneven number of participations from each site.

## Conclusion

This study presents the protocol of a two-arm waitlist-control randomized controlled trial study for the examination of a digital single-session growth mindset intervention for university students’ mental health. The current study will provide pioneer evidence for the feasibility of implementing tailored, easy-access, scalable, and brief digital interventions among university students in Chinese contexts.

## Trial status

The protocol version is 1.0, and the version date is May 20, 2023. Recruitment started from on April 21, 2023, and the approximate recruitment completed date is August 31, 2023.

### Supplementary Information

**Additional file 1.** SPIRIT Checklist for Trials.

## Data Availability

Not applicable.

## Abbreviations

SIGMA: Single-session Intervention on Growth Mindset for adolescents
U-SIGMA: Single-session Intervention on Growth Mindset for university students
MDASS: Mindset of Depression, Anxiety, and Stress Scale
TFA: Theoretical framework of acceptability
ACQ-EC: Emotion Control subscale of Anxiety Control Questionnaire
ATSPPH: Attitude toward Seeking Professional Psychological Help Scale
WEMWBS: Warwick-Edinburgh Mental Well-being Scale
GPAQ: Global Physical Activity Questionnaire
GAD-7: Generalized Anxiety Disorder-7
PHQ-9: Patient Health Questionnaire-9
PPS: Perceived Stress Scale
